# Pedagogic Strategies and Contents in Medical Writing/Publishing Education: A Comprehensive Systematic Survey

**DOI:** 10.3390/ejihpe14090165

**Published:** 2024-09-02

**Authors:** Behrooz Astaneh, Ream Abdullah, Vala Astaneh, Sana Gupta, Romina Brignardello-Petersen, Mitchell A. H. Levine, Gordon Guaytt

**Affiliations:** 1 Department of Health Research Methods, Evidence and Impact, Faculty of Health Sciences, McMaster University, Hamilton, ON L8S 4K1, Canada; gupts8@mcmaster.ca (S.G.); brignarr@mcmaster.ca (R.B.-P.); levinem@mcmaster.ca (M.A.H.L.); guyatt@mcmaster.ca (G.G.); 2 Department of Laboratory Medicine and Pathobiology, University of Toronto, Toronto, ON M5S 1A1, Canada; ream.abdullah@mail.utoronto.ca; 3 Faculty of Kinesiology and Health Sciences, York University, Toronto, ON M3J 1P3, Canada; vastaneh@cmcc.ca; 4 Department of Medicine, Division of Clinical Pharmacology and Toxicology, McMaster University, Hamilton, ON L8S 4K1, Canada; 5 Department of Medicine, McMaster University, Hamilton, ON L8S 4K1, Canada; 6 MAGIC Evidence Ecosystem Foundation, 0456 Oslo, Norway

**Keywords:** systematic review, medical writing, publishing, workshop, education

## Abstract

Workshops or training sessions on medical writing and publishing exist worldwide. We aimed to evaluate published articles about such workshops and examine both the content and teaching strategies employed. We searched ISI Web of Science, Ovid EMBASE, ERIC, Ovid Medline, and the grey literature. We considered no language, geographical location, or time period limitations. We included randomized controlled trials, before–after studies, surveys, cohort studies, and program evaluation and development studies. We descriptively reported the results. Out of 222 articles that underwent a full-text review, 30 were deemed eligible. The educational sessions were sporadic, with researchers often developing their own content and methods. Fifteen articles reported teaching the standard structure of medical articles, ten articles reported on teaching optimal English language use for writing articles, nine articles discussed publication ethics issues, and three articles discussed publication strategies to enhance the chance of publication. Most reports lacked in-depth descriptions of the content and strategies used, and the approach to those topics was relatively superficial. Existing workshops have covered topics such as the standard structure of articles, publication ethics, techniques for improving publication rates, and how to use the English language. However, many other topics are left uncovered. The reports and practice of academic-teaching courses should be improved.

## 1. Introduction

Publishing the findings of medical research is crucial, and doing so effectively requires expertise and confidence in both medical writing and adherence to established standards [1,2,3]. Publishing is the final step of research that transforms years of work into valuable knowledge accessible to the wider scientific community [4,5,6]. Success in medical publishing is not merely a personal validation; it is a key metric for career advancement in health science academia.

Publishing well-conducted research requires considering elements beyond the research itself. Authors must navigate specific requirements, including adherence to guidelines for structure released by related organizations, including the International Committee of Medical Journal Editors (ICMJE) [7,8]; tailoring content to specific article types with established checklists [9,10,11]; identifying the target audience; choosing the most suitable journal [12]; using clear and concise English for effective communication [13,14]; interacting effectively with editors and reviewers who play crucial roles in the publication process [15]; and understanding and following publication ethics considerations [16,17,18,19]. Post-graduate medical education often overlooks these crucial skills, leaving a gap that targeted workshops can effectively bridge by equipping researchers with the knowledge and tools needed to successfully navigate the publication landscape [20,21].

Health-professional educators use a variety of teaching techniques, including inquiry-based learning [22] and case-based learning, which have proven successful in particular settings [22]. None, however, have been formally tested in medical writing and publishing settings and, in these settings, the educational approach is often unsystematic, often relying primarily on the presenters’ personal experiences, and thus potentially neglecting the broader landscape of best practices and evidence-based guidance [23]. This approach risks inconsistency and subjectivity, potentially hindering participants’ acquisition of comprehensive knowledge and skills. Despite reported improvements in participants’ practical skills through some workshops [21,24,25,26], the extent of learning remains unclear. The scarcity of evaluations regarding content and educational strategies highlights a critical knowledge gap in this regard [20].

Although publishing workshops and training sessions are potentially important for improving medical writing, there is a lack of systematic analyses of their content and teaching strategies. This systematic review aims to fill this gap by evaluating published articles on such workshops and training sessions, examining both the teaching content and the knowledge transfer strategies employed.

Unlike scoping reviews that have broader research questions, systematic surveys address a more targeted research question. As we planned to focus only on the content and strategies used in such workshops, we chose a systematic survey methodology. We use the term systematic survey to differentiate it from systematic reviews of clinical areas [27,28].

This review is part of a larger project investigating various aspects of these workshops and medical journalology, leveraging a shared search strategy and database review. Findings related to the workshops’ impact [29] and qualitative analyses of participant experiences are presented in separate publications.

## 2. Materials and Methods

Although no reporting guideline is fully applicable to the nature of this systematic survey, where appropriate, we followed the PRISMA guideline [30].

### 2.1. Types of Studies

To comprehensively analyze the content and educational strategies employed in medical writing and publishing education, we included all studies that examined the workshops’ content and delivery from a diverse range of study designs, including randomized controlled trials (RCTs), cohort studies, before–after studies, surveys, and program evaluation and development studies. We included articles reporting any types of workshops, in-person or virtual, short vs. longer, or any training sessions in this regard, such as writing seminars. Articles reporting only the impact of the workshops, or a qualitative evaluation of participants’ experiences were excluded.

### 2.2. Types of Participants

We incorporated all the types of studies mentioned above, encompassing participants such as graduate students, medical students, post-graduate medical trainees, faculty members, and other willing adult participants who wanted to learn the skill of medical writing.

### 2.3. Search Methods for Identification of Studies

#### 2.3.1. Electronic Searches

We conducted a comprehensive search on Ovid EMBASE from inception to October 2022, using the search terms outlined in File S1. Additionally, we searched Ovid Medline from inception to October 2022, as detailed in File S2. A librarian assisted in selecting keywords and executing searches across the relevant databases. We employed database-specific keywords for the search, Mesh terms for Medline, and Emtree terms for the Embase search. We also searched the ISI Web of Science database using the specified keywords (File S3). Considering its specialized focus on medical education, another librarian who was well-versed in navigating the ERIC database participated in the search of this database.

We did not restrict our search by language, time period, or geographical location.

#### 2.3.2. Searching Other Resources

We reviewed the reference lists of the articles we found and looked into other sources, including the first 100 hits from a search of Google Scholar.

**Figure 1:**
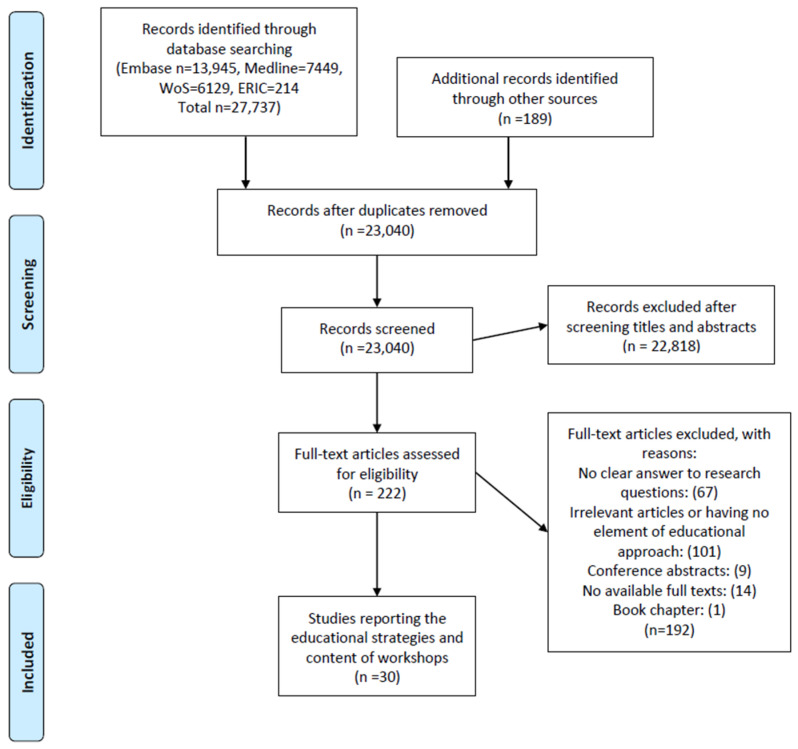
PRISMA flow diagram for pedagogic strategies and content in medical writing/publishing education.

To further ensure a comprehensive set of relevant articles, we reached out to experts in the field. We contacted journal editors and researchers in medical writing and publishing. We sent direct emails to those we knew and also posted a message in forums like the World Association of Medical Editors (WAME) and the Eastern Mediterranean Association of Medical Editors (EMAME). We asked for suggestions on articles not included in the databases we initially searched and information about any ongoing workshops with unpublished data. A PRISMA flow diagram shows the number of articles retrieved from different databases (Figure 1).

### 2.4. Data Collection and Analysis

#### 2.4.1. Selection of Studies

We created a team of reviewers (B.A., R.A., S.G., and V.A.). To create harmony among the reviewers, a set of 100 articles was selected as a calibration set, and during virtual meetings among the reviewers, instructions on how to screen the articles were finalized. The reviewers independently evaluated the articles and reached a consensus on the categories of results and the reproducible definition of each category. Pairs of reviewers independently screened the titles and abstracts of the studies using Covidence software, https://www.covidence.org/. For the studies that passed this initial screening, paired reviewers independently assessed the full texts for eligibility. If there were any disagreements between the reviewers, a third reviewer resolved them through a detailed discussion with the first two reviewers.

#### 2.4.2. Data Extraction and Management

We created a Google spreadsheet to collect data from the eligible articles. We conducted a virtual meeting among the four reviewers to discuss detailed instructions for data extraction. Subsequently, pairs of reviewers independently extracted data and resolved disagreements through discussion. To ensure consistency in judgments about data extraction, B.A. was a member of all reviewer pairs (i.e., B.A. and one of R.A., S.G., or A.A.). After piloting the process for one study, paired reviewers independently extracted data for the listed outcomes from each article. Discrepancies between the reviewers in terms of data were resolved through thorough discussions involving a third reviewer.

The collected data encompassed various aspects of the studies, including the workshop structure, objectives, workshop participant numbers, inclusion/exclusion criteria for participating in the workshops, participant recruitment methods, potential interventions, comparisons with interventions, the content of the teaching materials, the strategies used for teaching the materials, and anticipated outcomes. In case additional data were needed, we intended to reach out to the authors.

#### 2.4.3. Dealing with Missing Data

We planned to contact the authors of the studies for omitted important details regarding workshop content or teaching strategies.

### 2.5. Data Synthesis

#### Types of Outcome Measures

We organized the outcomes in the following categories:

The teaching content regarding the standard structure of medical articles.

The strategies to teach the standard structure of medical articles.

The teaching content regarding publishing standards and related ethical issues.

The strategies to teach the publishing standards and related ethical issues.

The teaching content regarding the optimal English language use in writing medical articles.

The strategies to teach optimal English language use.

The teaching content regarding improving the likelihood of publication and acceptance in prestigious journals.

The strategies to teach how to improve the likelihood of publication and acceptance in prestigious journals.

Our teams of independent reviewers evaluated the articles and agreed on the categories in the results that should align with the outcomes. We came up with reproducible definitions for each category and consulted with one another periodically during the data collection to see if modifications for the category descriptions were necessary. The educational content and strategies used to educate the participants for each outcome measure reported in each article were extracted and discussed, and decisions were made on how to present them descriptively by each team.

### 2.6. Methodological Quality of the Studies

All studies proved, simply from their study design, to provide only very low-certainty evidence regarding the impact of their teaching strategies, thus requiring no further assessment of risk of bias.

## 3. Results

Through exploration across various databases, we uncovered a total of 27,737 articles. Additionally, we retrieved 189 articles from other sources. Following the elimination of duplicates, we identified 23,040 unique reports. Subsequently, through screening titles and abstracts, we identified 222 articles for comprehensive full-text review. This process yielded 30 eligible articles reporting diverse educational strategies for workshop facilitation using different content presentations. Table S1 shows the 30 articles with topics covered by related facilitators. Figure S1 also shows different themes under each topic presented in the workshops. Table S2 shows all included articles and their main characteristics. Some studies reported more than one category or content. The PRISMA flow diagram shows the included articles. Some articles had missing data. We contacted the authors through their email addresses but received no response. Overall, the articles reported the areas that facilitators considered important to teach the participants as follows.

### 3.1. Structure of Medical Articles

Fifteen studies provided an overview of the standard structure of medical articles [26,31,32,33,34,35,36,37,38,39,40,41,42,43,44]. Two articles included information regarding the structure of case reports [26,41]. Most articles detailed in-person instruction, with only two reporting on virtual instruction [43,44].

Eight papers reported a single workshop setting [26,32,36,37,39,40,41,42], while the remaining seven articles provided training sessions over a more extended period [31,33,34,35,39,43,44].

All articles provided definitions of the most common sections of standard articles. Two articles explicitly mentioned the more minor sections of articles, such as the acknowledgments and bibliography sections [33,41]. In five articles, presenters provided students with real-life examples from previously published articles [26,32,38,39,44]. In one article, facilitators educated post-graduate students about the patterns found within sections of articles [39].

In four articles, presenters offered informative resources such as videos or references to other articles with recommendations on the correct structure [34,36,38,44]. Four workshops equipped participants with checklists to ensure all sections were complete and well organized [32,35,36,38]. Two articles reported using fillable templates or structured guidelines to instruct participants on the common structure of each section [32,37]. In one article, facilitators provided their own written piece to exemplify the sample format [35].

In four workshops, facilitators taught the popular acronym (IMRAD)—Introduction, Methods, Results, and Discussion—recommended by the ICMJE [26,32,33,41]. In two workshops, facilitators taught the variations in this acronym, such as IMRD and Abstract, Introduction, Methods, Results, and Discussion (aIMRAD) [39,44]. One article reported teaching students about the Summarize, Explain, Example, Review (SEER) acronym [35]. Another article reported using a broader strategy by teaching about US-style rhetorical organization of the main idea first, a body consisting of supporting details, and a conclusion reiterating the main point [31].

Six articles reported focusing on content selection [32,33,40,42,43,44]. These articles reported focusing on the types of information that should be included within each section, such as including clinical implications in the discussion section or duration of follow-up in the results [40]. Among these, two articles reported offering guidance on the appropriate proportions of text, figures, and tables [33,43]. In one workshop, facilitators detailed the correct placement of content within sections [44]. Three articles reported addressing transitioning between or within sections [37,39,42]. One article explicitly reported discussing subsections and their proper flow to novice researchers [44]. Teaching about headings and standard formatting was relatively uncommon [41]. Moreover, facilitators elucidated the art of crafting effective lead-in paragraphs in another workshop [42].

Educators used various techniques to apply the imparted knowledge in different educational sessions. Fourteen articles employed written hands-on exercises to assess participants [26,31,32,33,34,35,36,37,38,39,40,41,42,43]. Additionally, in one article, instructors had students present a verbal three-minute speech on the overall outline of their research project to help them conceptualize how an article is structured before beginning a writing assignment [31]. Another article reported giving students an article with the abstract removed and testing their ability to write a functional abstract [36]. In another article, facilitators concentrated on testing students with a range of multiple-choice questions [44].

Instructors commonly used feedback to help students or participants learn from their mistakes [32,34,35,37,39,42,44]. Four articles reported using peer feedback [34,37,39,42]. In one workshop, educators requested students to go back and write a new outline of their work if significant mistakes were found in the organization of their paper [35].

Two studies had participants work in groups [38,42]. The sizes of groups varied, from large group work [42] to both small and large groups [38].

Table 1 lists the articles reporting on the standard structure of articles discussed during the workshops, as well as their method of education and type of population.

### 3.2. Publishing Standards and Related Ethical Issues

Nine articles reported discussing various publication misconduct [25,45,46,47,48,49,50,51,52]. Of these, seven workshops aimed at teaching participants about publication ethics and assessing the impact of their teaching intervention [25,47,48,49,50,51,52]. The remaining articles reported surveying participants, including undergraduate students and postdoctoral fellows, to assess the impact of standard institutional training on real-life knowledge and experience with misconduct [45,46].

Workshop facilitators placed a significant focus on the ethics of authorship as they taught accurate reporting of authorship in five of these workshops [25,49,50,51,52]. The practice of ghostwriting and gift authorship were common ethical dilemmas described to participants by Trigotra et al. [51]. Gardner et al. distributed articles on the legal aspects and dishonest practices of guest- and ghostwriting for financial gain [47]. Only one article reported providing practical advice on how to manage co-authorship [49]. Three of these workshops focused on reporting ICMJE criteria to guide participants on how authorship should be selected [25,51,52].

In two workshops, educators also taught other criteria from a variety of different committees, including COPE, or mentioned guidelines such as CONSORT [25,51]. In one workshop, facilitators discussed more broad criteria for publishing, with an emphasis on common publishing guidelines that are usually present in journals in their field of research [49].

Three articles reported focusing on redundant or duplicate publications [48,50,51]. In two reports, instructors mentioned dual submission or salami publishing as other publication misconducts [47,51]. One article reported discussing a reference about the ethics of multiple submissions. Fake and predatory journals were also mentioned in this training writing intervention [47].

Six articles reported discussing ethical fraud. Three articles reported discussing the falsification of data [47,48,51], while all six articles reported plagiarism as a type of ethical fraud [25,47,48,50,51,52]. Rathore et al. focused on different types of plagiarism, including self-plagiarism, along with available anti-plagiarism software [25]. Other educators used real-life case-study examples of authors who had committed plagiarism [47]. Jawaid et al., employed a hands-on exercise to educate participants about plagiarism [52].

Three articles reported focusing on the importance of the full disclosure of conflicts of interest [25,50,51]. Gardner et al. discussed the issue of multiple submissions and how unethical and dishonest practices can ruin a career [47]. The purposeful exclusion of negative results that did not align with the authors’ hypothesis was also mentioned in another article [51]. Educators also discussed issues related to copyright and copyright protection in the workshops [25,50,52].

An important topic missing from medical writing and publishing workshops is image manipulation. Images are widely used in various types of medical articles, including pathology, radiology, or even basic-science articles. The temptation to manipulate images to showcase findings that authors would like to report is increasing, necessitating the education of researchers and teaching them how to avoid it [53].

Teaching methods across articles varied. Four articles reported using real-life case studies of publication misconduct [25,47,48,50]. Two studies focused on the importance of punishments received in these case studies as a motivating factor for their participants to avoid misconduct in the future [25,47]. Educators sometimes distributed books or articles on various ethical criteria to participants [47,50]. Only two articles reported employing the team-based learning (TBL) training method to invoke an active discussion on misconduct [48,50]. Table 2 shows the list of articles reporting on ethical issues discussed during the workshops or training sessions, as well as their method of education and type of population.

### 3.3. English Language Use in Writing Medical Articles

Ten articles reported focusing on the correct use of English in medical articles [32,35,36,39,41,47,49,54,55,56]. Of these, four articles reported teaching English to students who were enrolled in longer-duration courses [35,39,47,55]. And six reports focused on shorter teaching workshops [32,36,41,49,54,56].

In five articles, teaching methods consisted of lectures [32,36,39,47,49]. One study devoted 20 min at the end of each lecture so that participants could practice writing with the knowledge they had learned [32]. The other three opted for a more hands-on approach [35,54,55,56]. These studies began with writing exercises and allowed students to learn as they progressed through the workshops with the help of their peers and experienced instructors.

Six studies provided resources to students or readers to improve their writing [32,36,39,47,49,54]. Of these, three articles reported focusing on examples from published articles to illustrate both skilled and poor writing [32,39,54]. Other facilitators implemented hands-on writing exercises to teach participants in seven reports [35,36,39,47,54,55,56]. Some educators invited participants to work on drafts of medical papers they intended to publish [36,47,54,55]. Three articles reported creating new writing assignments to guide students [35,39,56], and six articles reported promoting peer feedback to guide reflections [32,39,41,47,54,56]. In two articles, educators used different rounds of resubmission-feedback to improve the writing assignments of students before they received their final grade [39,56].

Clarity and simplicity were emphasized as a key component of medical writing in four articles [32,36,41,49]. In a workshop for non-native English users, Heseltine provided multiple lists to illustrate how certain words and phrases could be simplified [36]. In three studies, facilitators explained these principles by encouraging writers to visualize the readers’ perspective as they wrote to improve their writing [41,49,54]. Some other educators explicitly recommended the use of short sentences as a technique to increase clarity [32,41]. Simple and less-complex words were also identified as a way to improve writing in two reports [41,47]. Three studies focused on the importance of structuring paragraphs and sentences [32,39,41]. For sentences, in one study, facilitators encouraged writers to keep their sentences to one idea [41], while other instructors focused on the correct placement of words within a sentence [39]. Instructors in one study helped to make sentences impactful using condensed and efficient wording [32]. Shankar and colleagues also recommended that writers focus on making each of their paragraphs convey a coherent and singular message and place extra attention on the first and last sentences of paragraphs [41]. Two other reports also emphasized paragraph clarity and ways to transition from paragraph to paragraph, along with the difference between discrete and structured paragraphing [32,39].

In other workshops, educators focused on correct tense or pronoun use [32,39,41], advising the use of active verbs versus passive ones [41,49] and discouraging the use of sexist or racist terms [41].

Technical components of writing proved a less popular theme across articles. While some educators discussed rhetorical writing [32,39,47], and the use of correct grammar and punctuation [32,39], one article reported discussing American, British, and Australian spelling [41]. Table 3 presents the articles reporting on English language use in writing medical articles discussed during the workshops or training sessions, as well as their method of education and type of population.

### 3.4. Improving the Likelihood of Publication

In three workshops, facilitators provided instructions to improve the likelihood of publication [21,57,58]. All three were workshops taught by experienced editors and faculty who regularly published papers within their field. One workshop focused on improving the likelihood of publication for nurses, midwives, and other similar professions [57]. In another workshop, the groups worked on a paper together and presented their results verbally to the entire cohort. Peers would then provide critical feedback, which would include ways to improve the paper so that the chances of publication could be increased [58].

All workshops focused on helping participants select a list of journals. One workshop focused on identifying journals that would lead to publication by reviewing journal requirements, journal interests, and published article types [21]. The other two workshops focused on selecting the most appropriate journals based on each article’s content [57,58].

In two workshops, educators provided links to articles with informative instructions and recommendations on achieving publication [21,58], in one of which facilitators also provided a workbook and action plan to help participants keep track of their publishing process [21].

Table 4 shows the list of articles reporting on ways to improve the likelihood of publications discussed during the workshops or training sessions, as well as their method of education and type of population.

## 4. Discussion

Overall, medical writing/publishing workshops or training sessions have focused on teaching the standard structure of medical articles, publication ethics, the use of the English language for writing articles, and, less frequently, a variety of tips to increase the chance of publication. The educational occasions occurred without an explicit schedule. Researchers used hands-on workshops, virtual training, and short-term courses. The participants included undergraduate and graduate students in different disciplines, medical residents, doctoral fellows, and faculty members. The topics were taught to both native and non-native English users.

### 4.1. Strengths and Limitations

The first strength of our research lies in its originality, as it represents the first article systematically delving into the content and educational strategies employed in workshops and training sessions for medical writing and publishing. This exposition thus provides a basis for evidence-based medical journalology. Another strength is the thorough search conducted across various databases, utilizing an extensive array of database-specific keywords. Additionally, the inclusion of the grey literature and a snowballing approach to identify all pertinent articles lend weight to our findings.

A major limitation of this study is that, because only a very small proportion of those conducting workshops publish papers describing the workshops, publications regarding workshops may be an unrepresentative sample of the workshops that are actually conducted. In addition, even of those who do publish descriptions of their workshops, the incomplete reporting of content and specific approaches utilized during teaching, particularly in the hands-on workshops, represent another limitation. These limitations hinder our capacity to provide robust recommendations regarding the optimal curriculum for such workshops. Despite this, our study provides valuable insights that augment the existing body of knowledge on medical writing and publishing education.

### 4.2. Relation to Prior Work

Using the standard structure for writing medical articles as recommended by the ICMJE is of paramount importance, a topic discussed in the majority of the articles we retrieved. However, beyond the original research articles that adhere to the recommended IMRaD format, various other article types are prevalent in medical and health-related journals, with case reports being particularly dominant. In our study, we identified only two articles providing guidance on writing case reports [26,41]. It is advisable to include instruction on writing other types of articles, such as various types of reviews, letters to editors, and so forth, in the curriculum of such workshops.

Educational strategies differ in their efficacy not only from one to another but also in the context in which they are implemented. For instance, efficacy may depend on educators’ and educatees’ characteristics, the content of the educational material being presented, associated evidence-based practices, and the types of feedback and evaluation undertaken.

Considering educational approaches in general, addressing cultural disparity and providing institutional support may enhance learning [59]. By recognizing cultural diversity and providing fair access to resources, educators can develop a healthy educational system that is useful for all trainees.

In terms of differences between approaches, active learning emphasizes that participants engage in the learning process [60]. This approach leads to more interaction between participants and educators, enhancing learning. Formative assessment is accompanied by the continuous assessment of students’ learning to provide feedback, which helps participants learn more quickly [61]. Differentiated instructions focus on diverse readiness levels and interests of participants, thus tailoring education to meet individual needs [62].

In our study, we found that educational strategies predominantly involved hands-on exercises and receiving feedback from facilitators or peers. The efficacy of a hands-on approach has been demonstrated in other disciplines, such as basic sciences [63]. Moreover, we have previously highlighted the positive impact of hands-on training on boosting confidence in medical writing among graduate students (currently under review in the PLOS One journal). Such hands-on training usually needs more resources, such as extra practice rooms and more facilitators, though.

Publication ethics is the most important consideration during the process of conducting and publishing research results [19]. No research, regardless of its methodological rigor, can be published if the related ethical issues are not considered; however, the standards are sometimes influenced by regional norms and regulations [64,65]. Reporting such ethical considerations might be overlooked when publishing the results of medical research [16]. Therefore, it is expected that educational approaches to medical writing and publishing comprehensively cover these topics.

Authorship has always been a matter of debate during research publication [18]. While authorship criteria based on the ICMJE recommendations and the importance of preventing ghost authorship and guest authorship have been discussed in some articles in our review [25,47,49,50,51,52], potential scenarios for legitimate changes in authorship during the publication process, or even after publication, based on the COPE flowcharts, were not addressed in any of these workshops [66,67,68,69].

Publication fraud, including data fabrication, data falsification, and plagiarism, is an unforgivable act that can be considered a crime by some authorities [70]. Plagiarism, as the most frequent form of fraud, and the use of software to detect it were discussed in some articles in our study [25,47,48,50,51,52]. However, data fabrication, as the most hazardous form of publication fraud, and the various methods to detect it, were not discussed in any of the retrieved articles [71]. This may be due to the need for statistical approaches for such detection, which requires relevant literacy.

Predatory publishing has threatened the integrity of ethical publishing for some years [72], affecting the rigor of publication practices. Therefore, it is worthwhile to discuss and teach how to avoid such journals in medical writing and publishing workshops. We found only one article reporting on the role of predatory publishing in our study [47].

Currently, English serves as the dominant language for academic medical publishing [73]. However, for those who are not native English speakers, composing medical articles in English poses a significant obstacle, leading to difficulties in getting published and a lower sense of confidence in comparison to native speakers [74,75]. Using simple and concise language is a critical aspect of conveying complex medical research to an international audience, particularly those who may not be native English speakers. While some of the articles we retrieved touched upon recommending simplicity and brevity in writing [32,41,49], it was not a predominant focus during the workshops. However, we have previously highlighted the importance of optimal English language use in enhancing confidence in medical writing [76]. Therefore, it is advisable to include instruction on using academic English language alongside teaching the standards of medical writing in relevant workshops or courses.

In addition to learning how to organize a standard medical article, use optimal language to convey the message, and consider related ethical issues, another important aspect to grasp is how to effectively publish such articles. Publication strategies were not a frequently discussed topic in the reports of workshops we retrieved [21,57,58]. However, understanding the nuances of journal selection and considering the requirements of the selected journal are crucial. It is worth noting that rejections from journals may not always stem from deficiencies in standard writing or suboptimal English usage; oftentimes, the culprit is selecting an inappropriate journal [77]. Instructing authors on how to approach journals’ instructions and thoroughly understand the scope of the journals can save valuable time by reducing the likelihood of rejection [78].

### 4.3. Implications for Educational Practice

In the absence of comprehensive, systematic academic courses on medical writing and publishing, workshops and short-term sessions are conducted worldwide. However, reports on the educational content and strategies used to teach participants, as well as their curricula, are often incomplete and vary among different workshops.

While topics such as the standard structure of articles, publication ethics, techniques for improving publication rates and acceptances to prestigious journals, and how to use the English language for writing articles have been relatively covered, most reports lack in-depth descriptions of the content and strategies used, and the approach to those topics are shallow and without covering all aspects of the topic.

Future workshops, including their detailed curricula and agendas, can benefit from considering the evidence gathered through this study. Agendas could cover the standard structure of medical articles (including IMRaD format, guidance on the appropriate proportions of text, figures, and tables), publication ethics (including publication fraud, redundant publications, multiple submissions), the use of English for writing articles (including writing with clarity and simplicity, using short sentences, choosing the correct tense or pronouns, using active versus passive verbs, writing with correct grammar and punctuation, and discouraging the use of sexist or racist terms), and finally using techniques to improve publication likelihood (including techniques of journal selection, avoiding predatory journals, and how to answer peer reviewers).

Another useful feature of the study is highlighting the topics that were ignored, including discussing changes in authorship after submission, image manipulation, multiple submissions, how to properly use checklists such as CONSORT or STROBE, specific terminology in their field, different types of medical articles published in medical journals, and nuances related to predatory journals, indexing systems, and scholarly metrics. Future workshops can also focus on exploring these topics. Artificial intelligence and its positive role in modern medical journalology, as well as its possible misuse and ethical concerns, is an emerging topic worth adding to future workshops [79,80].

Further steps and areas for research in this field include the quantitative evaluation of the impact of such workshops, and also a qualitative assessment of the experiences of the participants.

## 5. Conclusions

Facilitators should improve the reports and practice of academic teaching courses on medical writing and publishing. In addition to covering the standard structure of medical articles, publication ethics, the use of English for writing articles, and techniques to improve the likelihood of publication, we recommend that such teaching courses cover the above-mentioned neglected topics as well.

Standard academic writing and publishing courses with a defined curriculum covering all the aforementioned topics are recommended for graduate students, medical researchers, and faculty members who need to publish the results of their studies. This approach aims to address the current gaps in the field and enhance the effectiveness of educational initiatives related to medical writing and publishing.

## Figures and Tables

**Table 1:** List of articles reporting on the standard structure of medical articles discussed during the workshops or training sessions, as well as their method of education and type of population.

First Authors et al. (Ref.)	Year of Publication	Topic of Paper Structure Taught	Workshop Structure	Type of Population and (Number)
Hanson Diehl [35]	2007	SEER, structure analogies	Classroom course (3 sessions); storytelling exercise; individual and group exercises Sample format, checklist from instructor provided	Nursing graduate students (NR)
Fernandez et al. [33]	2018	IMRAD, types of abstracts, structure analogies, keywords, balance between text and figures, bibliography, acknowledgements, funding and competing interest sections	27 iterations of 2-day classroom course including lectures and individual and group exercises	Undergraduate and post-graduate degrees in health sciences (741)
Cameron et al. [32]	2009	IMRAD, content selection, how to build sections, examples from the literature	Workshop (18 h) lecture with time for participants to draft their own. Pairing with an editor/advisor	University of Texas postdoctoral fellows/trainees (>300)
Cameron et al. [31]	2011	US-style rhetorical organization	Program (11 weeks) with three sections: presentation, meeting and discussion, writing skills	International trainees (NR)
Wajekar et al. [26]	2018	IMRAD, examples from the literature	Interactive workshop on basic medical writing.	Post-graduate anesthesia residents (20)
Shah et al. [44]	2010	IMRAD, examples from the literature, content placement, content flow, roles of sections, argument flow, content selection, subsections role and framework, manuscript dissection, errors, ideal vs. unideal writing	Simulation material with multiple-choice test questions and embedded resources	Novice researchers (14)
Pololi et al. [42]	2004	Lead-in paragraphs, content selection	Collaborative mentoring program (Nine 9 h sessions). Seventy-five minutes of six day-long sessions allocated to scholarly writing	Assistant professors (18)
Shankar et al. [41]	2010	IMRaD, eight-heading format, standard format of papers, conclusion, acknowledgement, order of section writing (abstract, methods, and results), double spacing, key words, sequential order, narrative structure, literary rules for sections	Workshop (1 day)	Participants from institutions in Nepal, Malaysia, New York (49)
Malki et al. [40]	2003	Logic sequence, content selection, order of section writing	Workshop (1 day). Lecture and group discussions on case study	NR (21)
Li et al. [39]	2020	AIMRaD, patterns within sections, examples from the literature, syntactic structure, cohesion links	Semester-long writing for publication course	Year 1 doctoral students (25)
Li et al. [38]	2018	Examples from the literature, common errors	Workshop (3 h) with challenges, group exercises, and resources	Pediatric faculty and fellows in-training (33)
Jernigan et al. [37]	2014	Explaining sections with Freirean tradition methods, targeted questions/prompts, transitions	Pre-conference workshop (1 day) for integrating Indigenous and academic evaluation methods; data analysis workshop (4 days); scientific writing workshop (5 days)	Native American health professionals (9)
Griegel et al. [34]	2022	Section content and characteristics	Course; 3 modules (introduction, writing and feedback, presentation and defense training) with resources	Doctoral students of human/dental medicine (105)
Seres et al. [43]	2022	Balance between text and figures, content selection	Classroom-based course (2 days); virtual or in-person	NR
Heseltine [36]	2013	Proportions of text in sections, Bradford Hill criteria	Multiple iterations of a 3-day workshop; literature search/journal selection, group discussions, and informative resources	Non-native English speakers (>2000)

IMRAD/IMRaD—Introduction, Methods, Results, and Discussion. AIMRaD—Abstract, Introduction, Methods, Results, and Discussion. SEER—Summarize, Explain, Example, Review. IMRD—Introduction, Methods, Results, Discussion.

**Table 2:** List of articles reporting on ethical issues discussed during the workshops or training sessions, as well as their method of education and type of population.

First Authors et al. (Ref.)	Year of Publication	Topic of Publication Ethics Taught or Discussed	Workshop Structure	Type of Population and (Number)
Barrett et al. [46]	2005	Authorship guideline use and responsibility, copyright, conflict of interest, duplicate publications	Mandated RCR training (no further detail)	NIH-funded F32 postdoctoral fellowship awardees (423)
Abbott et al. [45]	2020	Limitations in authorship and publication practice knowledge	Authorship training (no further detail)	STEM Faculty members, Graduate and undergraduate students from USA (42)
Gardner et al. [47]	2018	Ghostwriting, multiple submissions, fabricated data, fake journals, plagiarism, ethical violations and punishments	Structured writing program (seminars, weekly writing group, discussions, one-on-one meetings) Informative resources given to attendees	Subgroup of PhD and MD/PhD students from the LLU-NIH IMSD program (6)
Trigotra et al. [51]	2019	Ghostwriting, honorary/gift authorship, plagiarism, salami publications, data manipulation, conflict of interest, omitting negative results, informed written consent, regulatory bodies and various guidelines (ICMR, ICMJE, COPE, ORI, CONSORT)	Educational lecture and survey	Medical, dental, physiotherapy, and nursing post-graduate students from four colleges of MMU (143)
Rathore et al. [25]	2018	ICMJE, unethical authorship and publishing, conflict of interest, copyright issues, informed consent, plagiarism, punishments	3 workshops (3 h each) and survey	Medical students and faculty members of Lahore Medical College (80)
Ju [48]	2009	Animal research ethics, fabrication, falsification, plagiarism, duplicate publication, authorship misconduct, IRB organization and maintenance	Tutor training course with TBL learning method (8 h) and survey	8 faculty and 3 staff at Hallym University (11)
Kim [50]	2008	Research misconduct, conflict of interest, copyright protection, plagiarism, authorship, duplicate publication	Course (4 h) with TBL learning method and survey Textbook provided	Physicians and basic medicine students at Hallym University (19)
Jawaid et al. [52]	2011	ICMJE authorship criteria, plagiarism, copyright	Four hands-on workshops (5 h each) and survey	Consultants, residents, house officers, medical students, and research associates from Pakistan (120)
Katsakhyan et al. [49]	2022	Co-authorship and authorship responsibility	Four virtual lectures (6 weeks) and survey	Pathology residents (MD and MD/PHD) at the University of Pennsylvania (27)

RCR—Responsible Conduct of Research. NIH—National Institutes of Health. STEM—Science, Technology, Engineering, and Mathematics. LLU-NIH IMSD—Loma Linda University Initiative funded by NIH for Maximizing Student Development. ICMR—Indian Council of Medical Research. ICMJE—International Committee of Medical Journal Editors. COPE—Committee on Publication Ethics. ORI—Office of Research Integrity. CONSORT—Consolidated Standards of Reporting Trials. MMU—Maharishi Markandeshwar University. IRB—Institutional Review Board. TBL—Team-based learning.

**Table 3:** List of articles reporting on English language use in writing medical articles discussed during the workshops or training sessions, as well as their method of education and type of population.

First Authors et al. (Ref.)	Year of Publication	Topic of Optimal Language Use Taught	Workshop Structure	Type of Population and (Number)
Salamonson et al. [56]	2010	Structure, feedback, and revision	Workshop (4 days)	First-year nursing students in Australia randomized to intervention (59) and control (47)
Katsakhyan et al. [49]	2022	Nominalization and action, clarity, prioritizing the reader	Four virtual lectures (6 weeks) and survey Educational materials provided	Pathology residents (MD and MD/PhD) at the University of Pennsylvania (27)
Shankar et al. [41]	2010	Active vs. passive voice, sentence length, simple language, clarity, prioritizing the reader, pronouns, structure, discriminatory language, regional spelling variance	Workshop (1 day)	Various participants from Nepali, Malaysian, and one New York institution (47)
Cameron et al. [32]	2009	Sentence length, clarity, tense use, structure, discrete paragraphing, grammar and punctuation, transitions, rhetoric, feedback	Workshop (3 weeks) 3 modules (6 h each) Handbook provided	Native- and non-native-speaking trainees and GME fellows at the University of Texas M. D. Anderson Cancer Center (46)
Gardner et al. [47]	2018	Rhetoric, precision, simple language feedback, grammar	Structured writing program (seminars, weekly writing group, discussions, one-on-one meetings) Informative resources provided	Subgroup of PhD and MD/PhD students (6)
Osman et al. [55]	2022	Feedback, grammar, and punctuation	Course (writing workshops, review article assignments, peer review sessions, journal club)	Fourth-year pharmacy students at Qatar University (NR)
Li et al. [39]	2020	Feedback, tense use, grammar and punctuation, structure, rhetoric, deductive and inductive teaching, word choice, useful phrases	Research writing course (assignments, lectures) Informative resources provided	Year 1 PhD students (55)
Hanson Diehl [35]	2007	Contextual topics, structure, elemental and higher-level writing	Beginner graduate course (assignments, lectures)	Graduate nursing students (NR)
Kulage and Larson [54]	2016	Prioritizing the reader, feedback, clarity, structure	Workshop (15 h across one semester) Informative resources provided	Students, post-doctoral fellows, and faculty (21)
Heseltine [36]	2013	Clarity, structure, simple language	Workshop (3 days) Informative resources provided	Non-native english speakers (2000)

GME—not specified by authors.

**Table 4:** The list of articles reporting on ways to improve the likelihood of publications discussed during the workshops or training sessions, as well as their method of education and type of population.

First Authors et al. (Ref.)	Year of Publication	Topic of Publication Strategies Taught	Workshop Structure	Type of Population (Number)
Steinert et al. [21]	2008	Target journals for publication, journal-specific requirements	Workshop (peer writing groups, group evaluation) Resources provided	Undergraduate/Post-graduate program directors and course coordinators (20)
Sridhar et al. [58]	2009	Case report requirements, publication resources, target journals for publication	Workshop (presentation and group sessions)	Clinician educator, residents, fellows, medical students (214)
Richardson and Carrick-Sen [57]	2011	Choosing appropriate journals, author guidelines	Workshop (structured and didactic, group interaction and discussion, and one-on-one mentorship) Resources provided	Nurses, midwives, occupational therapists (50)

## Data Availability

No new data were created or analyzed in this study. Data sharing is not applicable to this article.

## Supplementary Materials

The following supporting information can be downloaded at https://www.mdpi.com/article/10.3390/ejihpe14090165/s1, File S1: Ovid EMBASE search strategy; File S2: Ovid Medline search strategy; File S3: Web of Science search strategy; Table S1: Included articles and topics approached by facilitators in related workshops; Table S2: All included articles with their main characteristics; Figure S1: Topics presented in the workshops of the 30 included articles.
