# Supplementary material for: Pedagogic Strategies and Contents in Medical Writing/Publishing Education: A Comprehensive Systematic Survey
Source: Eur J Investig Health Psychol Educ. 2024 Sep 2;14(9):2491–508. doi: 10.3390/ejihpe14090165 (PMC11431838; doi:10.3390/ejihpe14090165)
Supplement: Supplementary file 1 [file ejihpe-14-00165-s001.zip › File S1.pdf]

Edit Search

Search Name: Final Final Embase search for SRs of publishing

Comment:

Save | Cancel

Set	Search Statement	Annotations	Insert	Edit	Delete
1.	Publishing*.mp.				
2.	medical journalism.mp.				
3.	journalology.mp.				
4.	exp publishing/				
5.	journalism, medical.mp.				
6.	medical literature/				
7.	medical writing*.mp.				
8.	manuscripts as topic/ or manuscripts, medical as topic/				
9.	manuscript*.mp.				
10.	medical publishing*.mp.				
11.	publication science.mp.				
12.	scholarly communication/				
13.	scholarly communication*.mp.				
14.	publication*.mp. or publication/				
15.	article structure.mp.				
16.	IMRAD.mp.				
17.	text structure.mp.				
18.	exp writing/				
19.	authorship.mp.				
20.	scientific writing*.mp.				
21.	medical writing*.mp.				
22.	publication ethics.mp.				
23.	ICMJE.mp.				
24.	((biomedical or bio-medical or medical or science or bioscience or bio-science or scientific or bio-scientific or health or healthcare or health care or scholarly) adj3 (journalis* or editing or writing or publishing)).ti,ab.				
25.	(authoring or authorship*).ti.				
26.	(authoring or authorship*).ab.				
27.	Research Report.mp.				
28.	journalism.mp.				
29.	Periodicals as Topic.mp.				
30.	publishing.ti,kw.				
31.	(Journalogy or Journalology).mp. [mp=title, abstract, heading word, drug trade name, original title, device manufacturer, drug manufacturer, device trade name, keyword, floating subheading word, candidate term word]				
32.	*writing/				
33.	*Manuscripts, Medical as Topic/				
34.	1 or 2 or 3 or 4 or 5 or 6 or 7 or 8 or 9 or 10 or 11 or 12 or 13 or 14 or 15 or 16 or 17 or 18 or 19 or 20 or 21 or 22 or 23 or 24 or 25 or 26 or 27 or 28 or 29 or 30 or 31 or 32 or 33				
35.	education/				
36.	*workshop/				
37.	workshop/ or workshop*.mp.				
38.	medical workshop*.mp.				
39.	medical writing workshop*.mp.				
40.	training/				
41.	training*.mp.				
42.	course*.mp.				
43.	seminar*.mp.				
44.	program development.mp. or program development/				
45.	35 or 36 or 37 or 38 or 39 or 40 or 41 or 43 or 44				
46.	experimental design.mp. or experimental design/				
47.	program evaluation.mp. or program evaluation/				
48.	exp program evaluation/				
49.	program evaluation.mp.				

50. before-after.mp.
51. interview/ or interview*.mp.
52. longitudinal studies.mp. or longitudinal study/
53. observational study.mp. or observational study/
54. cross-sectional study.mp. or cross-sectional study/
55. pilot study.mp. or pilot study/
56. survey*.mp.
57. questionnaire*.mp. or questionnaire/
58. 46 or 47 or 48 or 49 or 50 or 51 or 52 or 53 or 54 or 55 or 56 or 57
59. 34 and 45 and 58

Save Cancel

English Français Italiano Deutsch 日本語 繁體中文 Español 简体中文 한국어 About Us Contact Us Privacy Policy Terms of Use

© 2021 Ovid Technologies, Inc. All rights reserved. OvidUI_04.15.00.096, SourceID 206630d28626f3794d7f44308ce35cd7bc30c040
