# Supplementary material for: Pedagogic Strategies and Contents in Medical Writing/Publishing Education: A Comprehensive Systematic Survey
Source: Eur J Investig Health Psychol Educ. 2024 Sep 2;14(9):2491–508. doi: 10.3390/ejihpe14090165 (PMC11431838; doi:10.3390/ejihpe14090165)
Supplement: Supplementary file 1 [file ejihpe-14-00165-s001.zip › File S2.pdf]

Edit Search

Search Name: Final Final Medline search for SRs of publishing
Comment:

Save | Cancel

Set	Search Statement	Annotations	Insert	Edit	Delete
1.	publishing/ or journalism, medical/				
2.	Publishing*.mp.				
3.	Journalology.mp.				
4.	Medical Writing/				
5.	medical writing*.mp.				
6.	manuscripts as topic/ or manuscripts, medical as topic/				
7.	manuscript*.mp.				
8.	medical publishing*.mp.				
9.	publication science.mp.				
10.	Scholarly Communication/				
11.	scholarly publishing*.mp.				
12.	Publications/				
13.	Publication*.mp.				
14.	article structure.mp.				
15.	Journalism, Medical/				
16.	medical journalism.mp.				
17.	IMRAD.mp.				
18.	text structure.mp.				
19.	Authorship/				
20.	authorship.mp.				
21.	publication ethics.mp.				
22.	ICMJE.mp.				
23.	scientific writing*.mp.				
24.	writing/ or medical writing/				
25.	((biomedical or bio-medical or medical or science or bioscience or bio-science or scientific or bio-scientific or health or healthcare or health care or scholarly) adj3 (journalis* or editing or writing or publishing)).ti,ab.				
26.	(authoring or authorship*).ti.				
27.	(authoring or authorship*).ab.				
28.	Research Report/				
29.	Journalism/				
30.	Periodicals as Topic/				
31.	publishing.ti,kf.				
32.	(Journalogy or Journalology).mp. [mp=title, abstract, original title, name of substance word, subject heading word, floating sub-heading word, keyword heading word, organism supplementary concept word, protocol supplementary concept word, rare disease supplementary concept word, unique identifier, synonyms]				
33.	*writing/				
34.	*Manuscripts, Medical as Topic/				
35.	Education/				
36.	*workshop/				
37.	workshop*.mp.				
38.	medical workshop*.mp.				
39.	medical writing workshop*.mp.				
40.	training*.mp.				
41.	course*.mp.				
42.	seminar*.mp.				
43.	Program Development/				
44.	program*.mp.				
45.	experimental design.mp.				
46.	program evaluation.mp. or exp Program Evaluation/				
47.	before-after.mp.				
48.	Interview/				
49.	interview*.mp.				

50. Longitudinal Studies/
51. longitudinal study.mp.
52. Observational Study/
53. observational study.mp.
54. cross-sectional study.mp. or Cross-Sectional Studies/
55. pilot study.mp. or Pilot Projects/
56. "Surveys and Questionnaires"/
57. Survey*.mp.
58. questionnaire*.mp.
59. 1 or 2 or 3 or 4 or 5 or 6 or 7 or 8 or 9 or 10 or 11 or 12 or 13 or 14 or 15 or 16 or 17 or 18 or 19 or 20 or 21 or 22 or 23 or 24 or 25 or 26 or 27 or 28 or 29 or 30 or 31 or 32 or 33 or 34
60. 35 or 36 or 37 or 38 or 39 or 40 or 41 or 42 or 43 or 44
61. 45 or 46 or 47 or 48 or 49 or 50 or 51 or 52 or 53 or 54 or 56 or 57 or 58
62. 59 and 60 and 61

Save Cancel

© 2021 Ovid Technologies, Inc. All rights reserved. OvidUI_04.15.00.096, SourceID 206630d28626f3794d7f44308ce35cd7bc30c040
