# Supplementary material for: Pedagogic Strategies and Contents in Medical Writing/Publishing Education: A Comprehensive Systematic Survey
Source: Eur J Investig Health Psychol Educ. 2024 Sep 2;14(9):2491–508. doi: 10.3390/ejihpe14090165 (PMC11431838; doi:10.3390/ejihpe14090165)
Supplement: Supplementary file 1 [file ejihpe-14-00165-s001.zip › File S3.pdf]

Web of Science

Clarivate Analytics

The new Web of Science is here! CHECK IT OUT

Search History

Web of Science Core Collection

Set	Results	Save History / Create Alert · Open Saved History	Edit Sets	Combine Sets (AND / OR, Combine)	Delete Sets (Select All, Delete)
# 31	6,129	#30 AND #29 AND #28<br>*Indexes=SCI-EXPANDED, SSCI, A&HCI, CPCI-S, CPCI-SSH, BKCI-S, BKCI-SSH, ESCI, CCR-EXPANDED, IC Timespan=All years*	Edit	☐	☐
# 30	2,922,657	#27 OR #26 OR #25 OR #24 OR #23 OR #22<br>*Indexes=SCI-EXPANDED, SSCI, A&HCI, CPCI-S, CPCI-SSH, BKCI-S, BKCI-SSH, ESCI, CCR-EXPANDED, IC Timespan=All years*	Edit	☐	☐
# 29	894,324	#21 OR #20 OR #19 OR #18<br>*Indexes=SCI-EXPANDED, SSCI, A&HCI, CPCI-S, CPCI-SSH, BKCI-S, BKCI-SSH, ESCI, CCR-EXPANDED, IC Timespan=All years*	Edit	☐	☐
# 28	930,509	#17 OR #16 OR #15 OR #14 OR #13 OR #12 OR #11 OR #10 OR #9 OR #8 OR #7 OR #6 OR #5 OR #4 OR #3 OR #2 OR #1<br>*Indexes=SCI-EXPANDED, SSCI, A&HCI, CPCI-S, CPCI-SSH, BKCI-S, BKCI-SSH, ESCI, CCR-EXPANDED, IC Timespan=All years*	Edit	☐	☐
# 27	1,335,539	TS= survey*<br>*Indexes=SCI-EXPANDED, SSCI, A&HCI, CPCI-S, CPCI-SSH, BKCI-S, BKCI-SSH, ESCI, CCR-EXPANDED, IC Timespan=All years*	Edit	☐	☐
# 26	608,670	TS= experimental design*<br>*Indexes=SCI-EXPANDED, SSCI, A&HCI, CPCI-S, CPCI-SSH, BKCI-S, BKCI-SSH, ESCI, CCR-EXPANDED, IC Timespan=All years*	Edit	☐	☐
# 25	712,609	TS= questionnaire*<br>*Indexes=SCI-EXPANDED, SSCI, A&HCI, CPCI-S, CPCI-SSH, BKCI-S, BKCI-SSH, ESCI, CCR-EXPANDED, IC Timespan=All years*	Edit	☐	☐
# 24	613,987	TS= interview*<br>*Indexes=SCI-EXPANDED, SSCI, A&HCI, CPCI-S, CPCI-SSH, BKCI-S, BKCI-SSH, ESCI, CCR-EXPANDED, IC Timespan=All years*	Edit	☐	☐
# 23	26	TS= "before$after"<br>*Indexes=SCI-EXPANDED, SSCI, A&HCI, CPCI-S, CPCI-SSH, BKCI-S, BKCI-SSH, ESCI, CCR-EXPANDED, IC Timespan=All years*	Edit	☐	☐
# 22	7,361	TS= "before after"<br>*Indexes=SCI-EXPANDED, SSCI, A&HCI, CPCI-S, CPCI-SSH, BKCI-S, BKCI-SSH, ESCI, CCR-EXPANDED, IC Timespan=All years*	Edit	☐	☐
# 21	807,944	TS= training*<br>*Indexes=SCI-EXPANDED, SSCI, A&HCI, CPCI-S, CPCI-SSH, BKCI-S, BKCI-SSH, ESCI, CCR-EXPANDED, IC Timespan=All years*	Edit	☐	☐
# 20	343	TS= medical writing workshop*<br>*Indexes=SCI-EXPANDED, SSCI, A&HCI, CPCI-S, CPCI-SSH, BKCI-S, BKCI-SSH, ESCI, CCR-EXPANDED, IC Timespan=All years*	Edit	☐	☐
# 19	5,384	TS= medical workshop*<br>*Indexes=SCI-EXPANDED, SSCI, A&HCI, CPCI-S, CPCI-SSH, BKCI-S, BKCI-SSH, ESCI, CCR-EXPANDED, IC Timespan=All years*	Edit	☐	☐
# 18	96,593	TS= workshop*<br>*Indexes=SCI-EXPANDED, SSCI, A&HCI, CPCI-S, CPCI-SSH, BKCI-S, BKCI-SSH, ESCI, CCR-EXPANDED, IC Timespan=All years*	Edit	☐	☐
# 17	203,584	TS= writing*<br>*Indexes=SCI-EXPANDED, SSCI, A&HCI, CPCI-S, CPCI-SSH, BKCI-S, BKCI-SSH, ESCI, CCR-EXPANDED, IC Timespan=All years*	Edit	☐	☐
# 16	7,476	TS= scientific writing*<br>*Indexes=SCI-EXPANDED, SSCI, A&HCI, CPCI-S, CPCI-SSH, BKCI-S, BKCI-SSH, ESCI, CCR-EXPANDED, IC Timespan=All years*	Edit	☐	☐
# 15	6,030	TS= publication ethics<br>*Indexes=SCI-EXPANDED, SSCI, A&HCI, CPCI-S, CPCI-SSH, BKCI-S, BKCI-SSH, ESCI, CCR-EXPANDED, IC Timespan=All years*	Edit	☐	☐
# 14	298	TS= ICMJE<br>*Indexes=SCI-EXPANDED, SSCI, A&HCI, CPCI-S, CPCI-SSH, BKCI-S, BKCI-SSH, ESCI, CCR-EXPANDED, IC Timespan=All years*	Edit	☐	☐
# 13	17,466	TS= authorship*<br>*Indexes=SCI-EXPANDED, SSCI, A&HCI, CPCI-S, CPCI-SSH, BKCI-S, BKCI-SSH, ESCI, CCR-EXPANDED, IC Timespan=All years*	Edit	☐	☐
# 12	35,042	TS= text structure<br>*Indexes=SCI-EXPANDED, SSCI, A&HCI, CPCI-S, CPCI-SSH, BKCI-S, BKCI-SSH, ESCI, CCR-EXPANDED, IC Timespan=All years*	Edit	☐	☐
# 11	91	TS= IMRAD<br>*Indexes=SCI-EXPANDED, SSCI, A&HCI, CPCI-S, CPCI-SSH, BKCI-S, BKCI-SSH, ESCI, CCR-EXPANDED, IC Timespan=All years*	Edit	☐	☐
# 10	360	TS= medical journalism<br>*Indexes=SCI-EXPANDED, SSCI, A&HCI, CPCI-S, CPCI-SSH, BKCI-S, BKCI-SSH, ESCI, CCR-EXPANDED, IC Timespan=All years*	Edit	☐	☐
# 9	219,460	TS= article structure*<br>*Indexes=SCI-EXPANDED, SSCI, A&HCI, CPCI-S, CPCI-SSH, BKCI-S, BKCI-SSH, ESCI, CCR-EXPANDED, IC Timespan=All years*	Edit	☐	☐
# 8	263,513	TS= Publication*<br>*Indexes=SCI-EXPANDED, SSCI, A&HCI, CPCI-S, CPCI-SSH, BKCI-S, BKCI-SSH, ESCI, CCR-EXPANDED, IC Timespan=All years*	Edit	☐	☐
# 7	2,795	TS= Scholarly publishing*<br>*Indexes=SCI-EXPANDED, SSCI, A&HCI, CPCI-S, CPCI-SSH, BKCI-S, BKCI-SSH, ESCI, CCR-EXPANDED, IC Timespan=All years*	Edit	☐	☐
# 6	84,563	TS= *manuscript*<br>*Indexes=SCI-EXPANDED, SSCI, A&HCI, CPCI-S, CPCI-SSH, BKCI-S, BKCI-SSH, ESCI, CCR-EXPANDED, IC Timespan=All years*	Edit	☐	☐
# 5	154,496	TS= *publishing*<br>*Indexes=SCI-EXPANDED, SSCI, A&HCI, CPCI-S, CPCI-SSH, BKCI-S, BKCI-SSH, ESCI, CCR-EXPANDED, IC Timespan=All years*	Edit	☐	☐

# 4	3,817	TS= Medical publishing*<br>*Indexes=SCI-EXPANDED, SSCI, A&HCI, CPCI-S, CPCI-SSH, BKCI-S, BKCI-SSH, ESCI, CCR-EXPANDED, IC Timespan=All years*	Edit	☐	☐
# 3	264,136	TS= *Publication*<br>*Indexes=SCI-EXPANDED, SSCI, A&HCI, CPCI-S, CPCI-SSH, BKCI-S, BKCI-SSH, ESCI, CCR-EXPANDED, IC Timespan=All years*	Edit	☐	☐
# 2	5,432	TS= Medical writing*<br>*Indexes=SCI-EXPANDED, SSCI, A&HCI, CPCI-S, CPCI-SSH, BKCI-S, BKCI-SSH, ESCI, CCR-EXPANDED, IC Timespan=All years*	Edit	☐	☐
# 1	45	TS= *journalology*<br>*Indexes=SCI-EXPANDED, SSCI, A&HCI, CPCI-S, CPCI-SSH, BKCI-S, BKCI-SSH, ESCI, CCR-EXPANDED, IC Timespan=All years*	Edit	☐	☐
				○ AND ○ OR<br>Combine	Select All<br>✕ Delete

Clarivate
Accelerating innovation

© 2021 Clarivate Copyright notice Terms of use Privacy statement Cookie policy

Sign up for the Web of Science newsletter Follow us
