# Supplementary material for: Pedagogic Strategies and Contents in Medical Writing/Publishing Education: A Comprehensive Systematic Survey
Source: Eur J Investig Health Psychol Educ. 2024 Sep 2;14(9):2491–508. doi: 10.3390/ejihpe14090165 (PMC11431838; doi:10.3390/ejihpe14090165)
Supplement: Supplementary file 1 [file ejihpe-14-00165-s001.zip › Table S1.pdf]

Table S1: Included articles and topics approached by facilitators in related workshops

First authors et al (Ref)	Year of Publication	Structure of medical articles	Publishing standards/ Ethical issues	English language use	Improving publication likelihood
Steinert et al. [21]	2008	-	-	-	✓✓
Rathore et al. [24]	2018	-	✓✓	-	-
Wajekar et al. [25]	2018	✓✓	-	-	-
Cameron et al. [27]	2011	✓	-	-	-
Cameron et al. [28]	2009	✓✓	-	✓✓	-
Fernandez et al. [29]	2018	✓✓	-	-	-
Griegel et al. [30]	2022	✓✓	-	-	-
Hanson Diehl [31]	2007	✓	-	✓✓	-
Heseltine [32]	2013	✓✓	-	✓✓	-
Jernigan et al. [33]	2014	✓✓	-	-	-
Li et al. [34]	2018	✓✓	-	-	-
Li et al. [35]	2020	✓	-	✓✓	-
Malki et al. [36]	2003	✓✓	-	-	-
Shankar et al. [37]	2010	✓✓	-	✓	-
Pololi et al. [38]	2004	✓✓	-	-	-
Seres et al. [39]	2022	✓✓	-	-	-
Shah et al. [40]	2010	✓✓	-	-	-
Abbott et al. [41]	2020	-	✓✓	-	-
Barrett et al. [42]	2005	-	✓✓	-	-
Gardner et al. [43]	2018	-	✓✓	✓	-
Ju [44]	2009	-	✓✓	-	-
Katsakhyan et al. [45]	2022	-	✓	✓✓	-
Kim [46]	2008	-	✓✓	-	-
Trigotra et al. [47]	2019	-	✓✓	-	-
Jawaid et al. [48]	2011	-	✓✓	-	-
Kulage & Larson [50]	2016	-	-	✓✓	-
Osman et al. [51]	2022	-	-	✓✓	-
Salamonson et al. [52]	2010	-	-	✓✓	-
Richardson & Carrick-Sen [53]	2011	-	-	-	✓✓
Sridhar et al. [54]	2009	-	-	-	✓✓
