# Supplementary material for: Pedagogic Strategies and Contents in Medical Writing/Publishing Education: A Comprehensive Systematic Survey
Source: Eur J Investig Health Psychol Educ. 2024 Sep 2;14(9):2491–508. doi: 10.3390/ejihpe14090165 (PMC11431838; doi:10.3390/ejihpe14090165)
Supplement: Supplementary file 1 [file ejihpe-14-00165-s001.zip › Table S2.pdf]

Table S2: All included articles with their main characteristics

**Citation details (eg, author/s date, title, journal, volume, issue, pages**	**Type of evidence source**	**Methodology /approach /design**	**Country**	**Participant details**	**Context / Research question or study purpose:**
Hanson Diehl, S. Developing students' writing skills: an early intervention approach. Nurse Educ 2007, 32, 202-206, doi:10.1097/01.Nne.0000289377.06384.00	Standard structure & Optimal Language Use	Descriptive study of a prospective cohort	USA	Incoming graduate nursing students at the University of Hartford	Expand new graduate student knowledge on nursing and interdisciplinary literature and constructing theses & APA-style papers and describe the outcomes and process
Fernández, E.; García, A.M.; Serés, E.; Bosch, F. Students' satisfaction and perceived impact on knowledge, attitudes and skills after a 2-day course in scientific writing: a prospective longitudinal study in Spain. BMJ Open 2018, 8, e018657, doi:10.1136/bmjopen-2017-018657.	Standard structure	Prospective, longitudinal cohort with a pre and post assessment	Spain	Health science researchers in training or their first year (including undergraduate, postgraduate, medical residents & postdoctoral fellows)	Determine perceived long term impact & satisfaction and expand knowledge on scientific writing, editorial & peer review processes along with structure & content of standard scientific journals.
Cameron, C.; Deming, S.P.; Notzon, B.; Cantor, S.B.; Broglio, K.R.; Pagel, W. Scientific writing training for academic physicians of diverse language backgrounds. Acad Med 2009, 84, 505-510, doi:10.1097/ACM.0b013e31819a7e6d.	Standard structure & Optimal Language Use	Prospective cohort	USA	Post-doctoral fellows & trainees including both non-native and native english speakers at the University of Texas	Remedy the observed gap in writing knowledge & skills and gather objective outcome data to improve future iterations

Cameron, C.; Chang, S.; Pagel, W. Scientific English: a program for addressing linguistic barriers of international research trainees in the United States. J Cancer Educ 2011, 26, 72-78, doi:10.1007/s13187-010-0143-5	Standard structure	Descriptive	USA	International postdoctoral fellows and graduate students at M.D Anderson Cancer Center	Document the creation and delivery of a writing course along with providing suggestions & advice for other course creators
Wajekar, A.S.; Salgaonkar, S.V.; Chincholi, I.H.; Shetty, A.N. Impact of basic medical writing workshop on case report writing by post-graduate anaesthesia trainees: A pilot study. Indian J Anaesth 2018, 62, 502-508, doi:10.4103/ija.IJA_98_18	Standard structure	Prospective cohort with a pre and post assessment	India	Postgraduate anesthesia residents of Seth GS Medical College	Conduct a needs assessment analysis to identify areas of improvement in medical writing skills and identify if a workshop could remedy those weaknesses
Shah, J.; Rajgor, D.; Vaghasia, M.; Phadtare, A.; Pradhan, S.; Carvalho, E.; Pietrobon, R. WriteSim TCExam--an open source text simulation environment for training novice researchers in scientific writing. BMC Med Educ 2010, 10, 39, doi:10.1186/1472-6920-10-39.	Standard structure	Descriptive	Singapore	New graduate & postgraduate researchers	Document pilot experience and usability of a novel virtual simulation tool

Pololi, L.; Knight, S.; Dunn, K. Facilitating scholarly writing in academic medicine. J Gen Intern Med 2004, 19, 64-68, doi:10.1111/j.1525-1497.2004.21143.x.	Standard structure	Descriptive	USA	Primary Care Faculty (Assistant Professors) from 9 clinical departments at East Carolina University	Document the outcomes & experiences of a writing project in the Collaborative Mentoring program
Shankar, P.R.; Izham, M.I.M.; Piryani, R.M.; Subish, P. A one day workshop on scientific writing: Brief report. Australas Med J 2010, 1, 267-270, doi:10.4066/AMJ.2010.252.	Standard structure & Optimal Language Use	Descriptive	Nepal	Students from many different faculties & institutions from Nepal along with a student from Universiti Sains Malaysia, Malaysia & one student from Beth Israel Medical Center, New York.	Document the outcomes & experiences of a one-day writing workshop
Malki, A.A.; Al Bareeq, J.M.; Al Hilli, F.A. Evaluation of research writing workshop. Bahrain Medical Bulletin 2003, 25, 127-130	Standard structure	Descriptive questionnaire	Bahrain	NR	Document the outcomes of a questionnaire administered after a scientific writing workshop
Li, Y.; Ma, X.; Zhao, J.; Hu, J. Graduate-level research writing instruction: Two Chinese EAP teachers' localized ESP genre-based pedagogy. J Engl Acad Purp 2020, 43, 100813, doi:10.1016/j.jeap.2019.100813	Standard structure & Optimal Language Use	Case study	China	Chinese First Year Doctoral students	Document the methodology of two separate novice instructors

Li, S.-T.T.; Gusic, M.E.; Vinci, R.J.; Szilagyi, P.G.; Klein, M.D. A Structured Framework and Resources to Use to Get Your Medical Education Work Published. MedEdPORTAL 2018, 14, 10669, doi:10.15766/mep_2374-8265.10669.	Standard structure	Descriptive Cohort	USA	Pediatric faculty & fellows In-training from multiple different disciplines attending the Pediatric Academic Society Conference in 2016 and 2017	Document conceptual framework methodology, outcomes & experiences of teaching interventions in a one-day workshop
Jernigan, V.B.B.; Brokenleg, I.S.; Burkhart, M.; Magdalena, C.; Sibley, C.; Yepa, K. The implementation of a participatory manuscript development process with Native American tribal awardees as part of the CDC Communities Putting Prevention to Work initiative: Challenges and opportunities. Prev Med 2014, 67, S51-S57, doi:10.1016/j.ypmed.2014.01.027.	Standard structure	Descriptive cohort	USA	Native american health professionals from 1 of 3 communities (Cherokee Nation, Pueblo of Jemez, and the Great Lakes Inter-Tribal Council)	Document the implementation & translation of the scientific writing workshop

Griegel, S.; Kühl, M.; Schneider, A.; Kühl, S.J. Medical dissertation basics: analysis of a course of study for medical students. GMS J Med Educ 2022, 39, Doc26, doi:10.3205/zma001547.	Standard structure	Prospective cohort	Germany	Medical & Dental Doctoral Students	Measure satisfaction, acquisition of knowledge, & support of the Basics MED course
Serés, E.; Fernández, E.; García, A.M.; Vives-Cases, C.; Bosch, F. [Evaluation of competences in scientific writing after two different types of training courses: SCRIU-B study protocol]. Gac Sanit 2022, 36, 188-192, doi:10.1016/j.gaceta.2020.12.036.	Standard structure	Protocol	Spain	NR	Document the proposed methodology for an open and non-randomized experimental design study, which will instruct students and then evaluate satisfaction and writing skill improvement
Heseltine, E. Teaching scientific writing to non-native English speakers. Medical Writing 2013, 22, 13-16, doi:10.1179/204748012X13560931063591.	Standard structure & Optimal Language Use	Descriptive	France	Non-native english speakers	Document the outcomes & experiences of a 3 day workshop
Barrett, K.A.; Funk, C.L.; Macrina, F.L. Awareness of publication guidelines and the responsible conduct of research. Account Res 2005, 12, 193-206, doi:10.1080/08989620500217321.	Ethics	Longitudinal Cohort	USA	2002-2003 NIH-funded F32 postdoctoral fellowship awardees	Identify the effects of standard training on behaviour, awareness and attentiveness towards ethical conduct

Abbott, L.E.; Andes, A.; Pattani, A.C.; Mabrouk, P.A. Authorship not taught and not caught in undergraduate research experiences at a research university. Sci Eng Ethics 2020, 26, 2555-2599, doi:10.1007/s11948-020-00220-6.	Ethics	Longitudinal Cohort	USA	Wide variety of participants including all years of study, academic majors and multiple different ranks of faculty.	Identify how authorship decisions are made to establish an understanding of authorship negotiation
Gardner, S.A.; Salto, L.M.; Riggs, M.L.; Casiano, C.A.; De Leon, M. Supporting the Writing Productivity of Biomedical Graduate Students: An Integrated, Structured Writing Intervention. CBE Life Sci Educ 2018, 17, ar45, doi:10.1187/cbe.16-12-0350.	Ethics & Optimal Language Use	Retrospective Mixed Methods Analysis	USA	Non-native & Native PHD & MD/PHD speakers who completed the program previously & have graduated from the Loma Linda University Initiative	Identify if the objectives of the program were met, which included reduced writing anxiety, increased writing & writing skills, and writing confidence
Trigotra, S.; Jaiswal, S.; Mittal, A.; Bhardwaj, A. Impact of a publication ethics orientation program on the knowledge and attitude of postgraduate students of health sciences. J Clin Diagn	Ethics	Prospective cohort with a pre and post assessment	India	Postgraduate students from four colleges of MMU: MM Institute of Medical Sciences and Research (MMIMSR),	Identify the impact of the orientation program on students knowledge and attitude

Res 2019, 13, LC04-LC09, doi:10.7860/JCDR/2019/40008.12548.				MM College of Dental Sciences and Research (MMCDSR), MM Institute of Physiotherapy and Rehabilitation (MMIPR) and MM College of Nursing (MMCON)	
Rathore, F.A.; Fatima, N.E.; Farooq, F.; Mansoor, S.N. Combating Scientific Misconduct: The Role of Focused Workshops in Changing Attitudes Towards Plagiarism. Cureus 2018, 10, e2698, doi:10.7759/cureus.2698.	Ethics	Cross-sectional cohort	Pakistan	Final year medical students & faculty members of Lahore Medical College	Identify the impact of the assessment on reducing scientific misconduct
Ju, Y.-S. Evaluation of a team-based learning tutor training workshop on research and publication ethics by faculty and staff participants. J Educ Eval Health Prof 2009, 6, 5, doi:10.3352/jeehp.2009.6.5.	Ethics	Prospective Cohort	South Korea	Faculty members & staff at Hallym University	Describe the methodology of the team-based tutor training workshop & evaluation of the course by participants

Kim, S.Y. Students' Evaluation of a Team-based Course on Research and Publication Ethics: Attitude Change in Medical School Graduate Students. J Educ Eval Health Prof 2008, 5, 3, doi:10.3352/jeehp.2008.5.3.	Ethics	Prospective Cohort	South Korea	Graduate students in the medical school of Hallym University (working physicians & full-time students)	Describe methodology of the course & identify areas of improvement for future iterations
Jawaid, M.; Masood, Z.; Alam, S.N.; Jawaid, S.A. An analysis of interactive hands-on workshops on medical writing. J Pak Med Assoc 2011, 61, 66-70.	Ethics	Prospective Cohort with pre and post assessment	Pakistan	Consultants, Residents, House Officers, Medical Students, & Research Associates	Identify the impact of the workshop on the improvement of students knowledge & skills, and identify areas of improvement for future iterations
Katsakhyan, L.; Jacobson, A.M.; Budina, A.; Baloch, Z.W. Practical Scientific Writing and Publishing in Anatomic Pathology. Am J Clin Pathol 2022, 157, 451-456, doi:10.1093/ajcp/aqab144.	Ethics & Optimal Language Use	Prospective Cohort with pre and post assessment	USA	MDS & MD/PHDs at the University of Pennsylvania	Conduct a needs assessment analysis to identify areas of improvement and develop & test a curriculum suited to those needs
Salamonson, Y.; Koch, J.; Weaver, R.; Everett, B.; Jackson, D. Embedded academic writing support for nursing students with English as a second language. J Adv Nurs 2010, 66, 413-421,	Optimal Language Use	Randomized Control Trial	Australia	First year Nursing students with to low to medium english proficiency	Identify the impact of the workshop in improving academic writing skills

doi:10.1111/j.1365-2648.2009.05158.x.					
Osman, A.; Al-Badriyeh, D.; Hussain, F.N.; Riaz, S.; Elewa, H.; Mraiche, F. The design and implementation of an undergraduate health professional degree elective course on scientific writing, peer assessment, and critical appraisal. Curr Pharm Teach Learn 2022, 14, 765-772, doi:10.1016/j.cptl.2022.06.008	Optimal Language Use	Descriptive cohort	Qatar	Pharmacy students in their final year at Qatar University, majority were non-native english speakers	Document the course methodology & perceived impact & satisfaction
Kulage, K.M.; Larson, E.L. Implementation and Outcomes of a Faculty-Based, Peer Review Manuscript Writing Workshop. J Prof Nurs 2016, 32, 262-270, doi:10.1016/j.profnurs.2016.01.008.	Optimal Language Use	Descriptive Cohort	USA	Clinical & Research faculty members, research scientists, postdoctoral fellows & doctoral students	Document workshop methodology, along with perceived skill improvements and publication output
Steinert, Y.; McLeod, P.J.; Liben, S.; Snell, L. Writing for publication in medical education: the benefits of a faculty development workshop and peer writing group. Med	Improving Likelihood Of Publication	Prospective cohort with pre and post assessment	Canada	Faculty members (undergraduate or graduate course coordinators & program directors)	Eliminate writing barriers

Teach 2008, 30, e280-285, doi:10.1080/01421590802337120.					
Sridhar, A.R.; Willett, L.L.; Castiglioni, A.; Heudebert, G.; Landry, M.; Centor, R.M.; Estrada, C.A. Scholarship opportunities for trainees and clinician educators: learning outcomes from a case report writing workshop. J Gen Intern Med 2009, 24, 398-401, doi:10.1007/s11606-008-0873-9.	Improving Likelihood Of Publication	Prospective cohort with pre and post assessment	USA	USA, Canada, & Japanese clinical-educators, medical students, residents & fellows	Assess post-workshop learning outcomes & improvement of case report writing skills
Richardson, A.; Carrick-Sen, D. Writing for publication made easy for nurses: an evaluation. Br J Nurs 2011, 20, 756-759, doi:10.12968/bjon.2011.20.12.756	Improving Likelihood Of Publication	Prospective cohort	USA	Nurses, midwives & an occupational therapist	Eliminate writing barriers & increase nursing publication submission rates
