# Supplementary figures and images for: Pedagogic Strategies and Contents in Medical Writing/Publishing Education: A Comprehensive Systematic Survey
Source: Eur J Investig Health Psychol Educ. 2024 Sep 2;14(9):2491–508. doi: 10.3390/ejihpe14090165 (PMC11431838; doi:10.3390/ejihpe14090165)

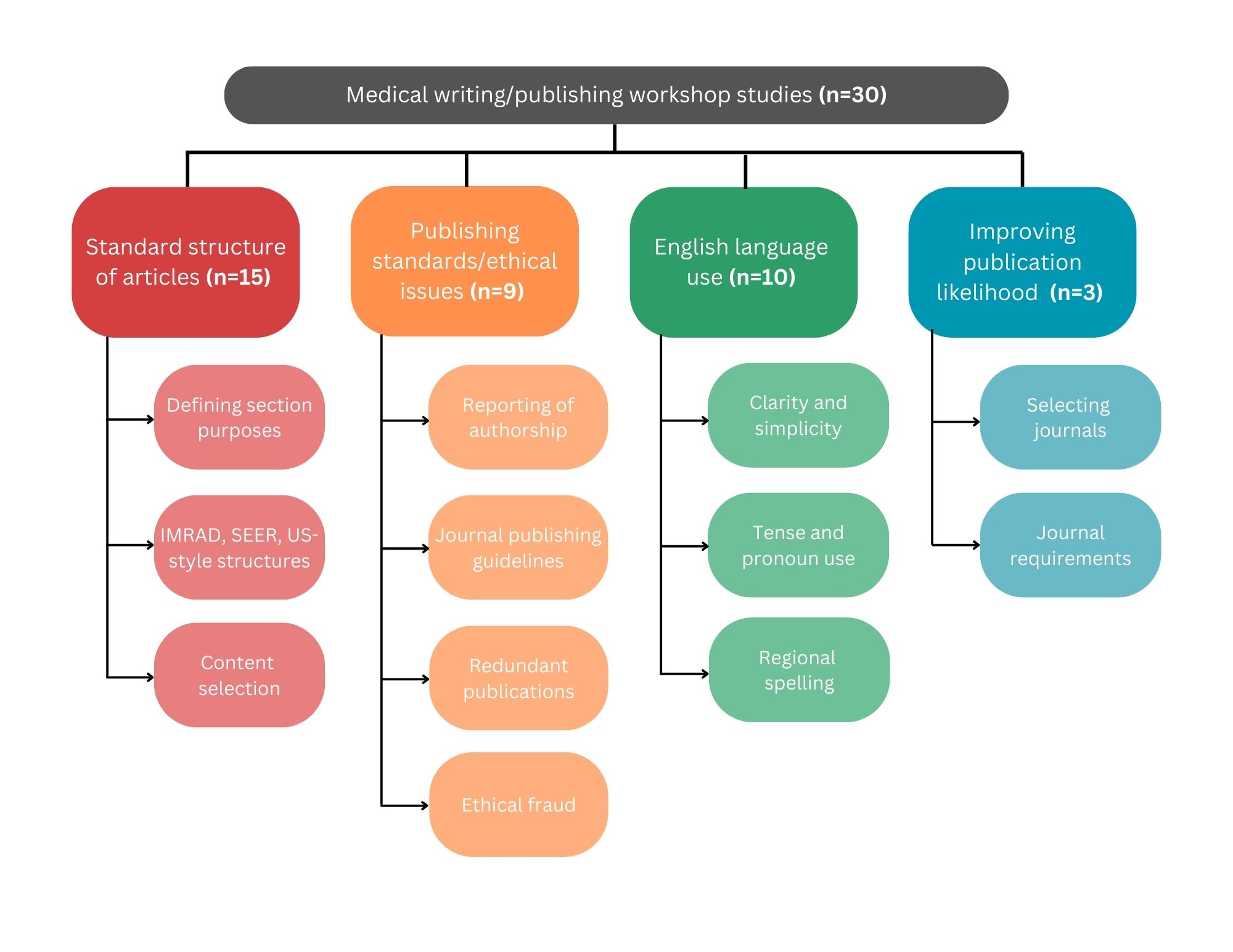

Supplement: Supplementary file 1 [file ejihpe-14-00165-s001.zip › Figure S1.jpg]
